# Development of Chitosan/Starch-Based Forward Osmosis Water Filtration Bags for Emergency Water Supply

**DOI:** 10.3390/membranes10120414

**Published:** 2020-12-11

**Authors:** Saiful Saiful, Maurisa Ajrina, Yusuf Wibisono, Marlina Marlina

**Affiliations:** 1 Chemistry Department, Faculty of Mathematics and Natural Science, Syiah Kuala University, Banda Aceh 23111, Indonesia; maurisaajrina62@gmail.com (M.A.); marlina@unsyiah.ac.id (M.M.); 2 Department of Bioprocess Engineering, Faculty of Agricultural Technology, Brawijaya University, Malang 65141, Indonesia; y_wibisono@ub.ac.id

**Keywords:** filter water bag, chitosan, *Dioscorea hispida*, starch, forward osmosis membrane, glutaraldehyde, emergency

## Abstract

A forward osmosis (FO) membrane was developed from a mixture of chitosan and *Dioscorea hispida* starch, cross-linked using glutaraldehyde. The cross-linked chitosan/starch membrane was revealed to have high mechanical properties with an asymmetric structure. The prepared membrane’s performance was investigated as an FO filter assembled in a polypropylene water filter bag and aluminum foil plastic. In order to study the FO process, brackish water was used as a feed solution, drawn using three types of solution (fructose, sucrose, and fructose/sucrose mixture, each with 3 M concentration). The maximum water flux (5.75 L/m^2^ h) was achieved using 3 M sucrose. The cross-linked membrane restrained the ions in the feed with a rejection factor value close to 100%. The water quality parameters were evaluated for the physical, chemical, and biological criteria, such as pH, salinity, conductivity, total dissolved solids (TDS), heavy metals, and *Escherichia coli* content. The water quality parameters for the FO-processed water met that set by the World Health Organization for drinking water. FO filter bags with cross-linked chitosan/starch membranes can be an option to produce drinking water during an emergency.

## 1. Introduction

Water is a natural resource that is essential for human life and supports various daily activities [1]. Water is the primary component of life and contributes to vital functions within the human body [2]. The demand for clean and fresh water, especially for consumption purposes, is expected to increase annually, along with the surge in human population and an increase in global water pollution [2]. For instance, global water consumption in the 1900s was 358 km^3^ per year and it increased five-fold to 1500 km^3^ per year in the 2000s. Water sources on earth can be obtained from seawater, spring water, groundwater, freshwater lakes, rivers, and the atmosphere. However, most available water sources in nature cannot be accessed and used directly as a source of clean and safe drinking water. Despite the abundance of the earth’s water sources, only about 1% of them can be consumed directly, while the other 97% are in the form of seawater, which is unsafe to consume directly [3]. Therefore, water is considered a significant world resource problem, along with food and energy [4].

Moreover, the demand for clean and fresh water during emergencies, increases compared to normal conditions due to the potential interruption to public utilities. Yet, the water qualities should be maintained to meet the established standards [5]. A more practical alternative method and tool is needed to provide clean drinking water during insurgencies, such as in disaster areas, conflict zones, water crises, and other emergencies. Membrane technology driven by applied pressure and osmotic gradients is suitable for use in emergencies [6]. The membranes commonly used are microfiltration (MF); followed by ultrafiltration (UF), nanofiltration (NF), reverse osmosis (RO), and forward osmosis (FO) [7,8]. Large contaminants in the range of 0.1–5 µm, such as bacteria, viruses, and protozoa in raw water can be removed using MF and UF membranes. Advantages of MF and UF membranes operating at relative pressures. Nanofiltration (NF) and reverse osmosis (RO) membranes were applied to remove smaller size contaminants (ionic components). NF membranes can effectively detect divalent ions, whereas RO membranes can reject monovalent ions. RO membranes are commonly utilized to produce clean and potable water from brackish and seawater. Meanwhile, the FO membrane is a membrane method currently developing with the driving force of the osmotic gradient, without the need to exert external pressure to force fluid flow across the membrane. For practicality in an emergency, forward osmosis (FO) membrane-based filter bag can be proposed as a potential alternative [5,9]. FO membranes are a water separation process that uses the difference in osmotic pressure to produce a water flow through the semipermeable membrane, for separation of water from the dissolved solute [10]. The process of purifying water with FO is carried out by utilizing an osmotic pressure gradient that draws solutes so that water from the feed solution (FS) passes through the semipermeable membrane towards the draw solution (DS) side [11]. Over the past few years, the FO method attracted much attention on both a laboratory and industrial-scale because FO provides many benefits, such as lower energy use, lower tendency for fouling, and better water purification results [12]. Due to its energy efficiency and simple instrumentation, the FO technology provides a great potential for water purification options during emergencies.

FO membranes are manufactured using polymeric materials, both synthetic and natural polymers. Previous studies showed that chitosan as a natural polymer can be used as a membrane for brackish water purification [5,9] and seawater desalination [13,14]. An initial study on the manufacture of drinking water bags using chitosan-based FO membranes was conducted [9]. The results showed that the chitosan-based FO membrane possesses a great potential in brackish water purification because it can produce water that is free of salt, metals, bacteria, and other dissolved materials, therefore, meeting the standards for drinking water. However, it was also reported that pure chitosan membranes are likely to be rigid, fragile, and not acid-resistant. A potential solution is to modify the chitosan membrane to create a better quality membrane and to overcome the limitation of the pure chitosan membrane [12].

The chitosan membrane can be modified through the addition of other polymers through a cross-linking method. Via the cross-linking method, the polymer becomes resistant to acids, thereby increasing its mechanical and chemical stability [15]. Natural polymers from non-food plants are widely used as a source of starch. *Dioscorea hispida* tubers are known as natural starch sources that are cheap and easily found in tropical regions [16]. The advantage of *D. hispida* is its high starch contents, which surpasses 70% yield [17]. *D. hispida* also contains crude protein around 3.6–9.8%. The fat content is relatively low at 1.99–9.36% and the ash content is at 0.29–1.24%. The main mineral is phosphorus, with a value of 11.7–46.9 mg/100g. The cyanide content in tubers is 379–739 ppm. However, this cyanide can be easily removed by washing, using water repeatedly. The *D. hispida*-based starch can be used for a variety of applications, including membrane preparation [18]. The membranes made from a mixture of natural materials can be used as an alternative, environment-friendly material with a great potential production and economic value. Glutaraldehyde is widely used as a cross-linking agent to produce composite films [19] and chitosan membranes [20]. Meanwhile, the properties of pure chitosan membranes that lead to a stiff form can be overcome by adding plasticizers. Glycerol is a commonly used plasticizer to make starch–chitosan mixture films [21] and starch–chitosan edible films [22].

In this study, FO membrane sheets were synthesized from chitosan–*D. hispida*-starch cross-linked by glutaraldehyde. The FO sheet was further applied to manufacture drinking water filtration bags. Membrane modification was done by referring to previous studies [18]. The modified membrane was applied to the drinking water bags made of polypropylene (PP) plastic and aluminum foil plastic. The FO filter water bag was evaluated in terms of its performance drinkability substance as draw solution, such as fructose, sucrose, and a mixture of both solutions. Brackish water was used as a feed solution, mimicking the water contamination that might be present in an emergency, while the FO process was intended to produce direct drinking water. The water quality parameters were evaluated based on the physical, chemical, and biological criteria by analyzing pH, salinity, conductivity, metal content (As, Cd, Cr, Cu, Fe, Hg, and Zn), and *Escherichia coli* bacteria content.

## 2. Materials and Methods

### 2.1. Materials

The chitosan was purchased from a local chitosan manufacturer that met international medical and food-grade standards, with an acetylation degree of up to 94 mol% (CV. Multiguna, Cerebon, Indonesia). Starch extracted from *D. hispida* tubers was used as a polymer mixture. Glacial acetic acid (Merck, Darmstadt, Germany) was used as a solvent. Sodium bisulfite (Merck, Darmstadt, Germany) was used to prevent browning in starch isolation. Sodium hydroxide was used to neutralize the acetic acid content during membrane cleaning. Fructose and sucrose (Merck, Darmstadt, Germany) were used as draw solutions. Silver nitrate (Merck, Darmstadt, Germany) was used in cyanide qualitative tests. Glycerol (Merck, Darmstadt, Germany) was used as a plasticizer. Glutaraldehyde (Merck, Darmstadt, Germany) was used as a cross-linking agent. Aluminum foil, packaging caps, and PP plastic were obtained from the local plastic store (Banda Aceh, Indonesia). The FO feed solution was brackish water obtained from the Krueng Aceh River and the dam water was obtained from the Limpok area, Aceh Besar District, Indonesia.

### 2.2. D. hispida-Based Starch Extraction

To extract the starch, *D. hispida* tubers were firstly peeled and washed thoroughly, using distilled water. Then, the clean the tubers were sliced into a smaller size and added with sodium bisulfite (1.12 g/L), followed by mashing with a crusher to obtain a tuber pulp. It was soaked into distilled water and squeezed using gauze. The filtrate was allowed to settle for 24 h to produce a precipitate. The water in the upper layer was slowly removed. Distilled water was added to dissolve the precipitate, followed by a filtration using a Buchner vacuum. The tubers were washed several times to remove the cyanides. The successful removal was tested using 2 M silver nitrate, where the addition did not change the color of the mixture solution into brown. The precipitate was oven-dried for 24 h at 70 °C. The dried precipitate was sieved with a 100-mesh sieve to obtain starch flour [16]. 

### 2.3. Membrane Preparation

The membranes were prepared using a chitosan/starch (2:1) mixture. To obtain the mixture, starch paste and chitosan solution were first produced. To produce a starch paste, starch flour was suspended in distilled water and stirred evenly. The mixture was then heated at 75–80 °C for 10–15 min, to reach gelatinization. Meanwhile, the chitosan was dissolved in a 1% (*v*/*v*) acetic acid solution and stirred to obtain a chitosan solution. The chitosan solution and starch paste were mixed and stirred at 75–80 °C for ±10 min, and then it was left at room temperature. Afterward, 5.6 × 10^−5^ mol glutaraldehyde and 0.4% (*v*/*v*) glycerol were added. The solution was stirred for ±20 min until homogeneous, cast on a ceramic plate, and oven-dried at ±30 °C. Once dried, the membrane was removed from the mold, washed with 1% (*w*/*v*) NaOH, and rinsed with distilled water. Finally, the membranes were air-dried at room temperature [18]. The membrane was then assembled into a filter water bag and tested for its performance in FO.

### 2.4. Drinking Water Bags Fabrication

The fabrication of drinking water bags was done by using the prepared membranes, PP plastic, and aluminum foil. Two pieces of PP plastic and one sheet of aluminum foil were cut to a designated size (12.5 × 19 cm^2^). In the middle of one of the PP plastic pieces, a hole with a size of 6 × 10.5 cm^2^ was made. After that, the FO membrane (8 × 13 cm^2^) was attached to a PP plastic that was given a hole. The membrane was attached using VHB double-sided foam tape. Three layers consisted of PP plastic (1), PP plastic with attached FO membrane (2), and aluminum foil (3); all layers were assembled from top to bottom, sequentially. The bags were glued by lamination, using a sealing machine. Finally, the lid was installed on the front and backside of the bag.

### 2.5. Assessment of Drinking Water Bags

Three different DSs (fructose, sucrose, and fructose/sucrose mixture, each with 3 M concentration) were used, respectively, to assess the FO membrane performance in the drinking water bag. Brackish and dam water was used as an FS. Drinking water bags were filled with 100 mL DS through the front opening. Then, the drinking water bag’s back opening was filled with 200 mL FS to initiate the FO process (up to 1 h). The water flux (L/m^−2^ h^−1^) and rejection factor (%) were then calculated using Equations (1) and (2), respectively.

(1) $$Water\;flux=\frac{\Delta V}{A\;\Delta t}$$

(2) $$Rejection\;factor=1-\frac{C_{p}}{C_{f}}\times 100\%$$

where ∆*V*, ∆*t*, and *A* represent the volume of FO-processed water (L), the FO duration (hour), effective membrane surface area (m^2^). Meanwhile, *C_p_* and *C_f_* are solute concentrations (TDS) in the processed water and FS, respectively.

### 2.6. Foward Osmosis Water Product Analysis 

The water quality produced was determined by pH, salinity, conductivity, metal content, and *E. coli bacteria* content. The pH, salinity, and TDS level was measured using pH meter CT-6022 (Shenzhen Kedida Electronics Co. Ltd., Shenchen, China), Salinity Meter SA287 (Guangzhou 3win Electronic Technology Co. Ltd., Guanhzhou, China), and conductivity meter WTW LF320 (Wissenschaftlich-Technische-Werkstätten GmbH, Weilheim, Germany), respectively. Metal contents (As, Cd, Cr, Cu, Fe, Hg, and Zn) were measured using atomic absorption spectrometer (AAS) Shimadzu 5960A (Kyoto, Japan). Finally, the *E. coli* content was determined using the most probable number (MPN) method [23].

## 3. Results and Discussion

### 3.1. D. hispida-Based Starch Extraction

The isolation of starch was carried out using distilled water as a solvent and the solubility of starch was assisted by the addition of sodium bisulfite into the *D. hispida* slurry. Sodium bisulfite also helps to prevent the browning of starch and activation of bacteria. The process of starch isolation requires repeated washing with water to remove cyanide toxin compounds. Previous studies reported that the cyanide toxin content, in the form of HCN, can reach 700 mg/kg [16]. It can be removed by repeated washing. As a result, the starch was free of cyanide toxin, qualitatively marked by the unchanged color after the addition of 1 M silver nitrate addition. Dried *D. hispida*-based starch powder obtained in this study had a yield of 7%.

### 3.2. Forward Osmosis Membrane Preparation

The membrane prepared in this research was based on the optimum condition in our previously published report [18]. During the preparation of starch paste, prior to the making of chitosan/starch mixture, the gelatinization process caused the starch granule crystals to absorb water, swell, then break, and dissolve in water. To cross-link the chitosan and starch, glutaraldehyde was used, where its excessive addition could promote the formation of aggregates, resulting in an inhomogeneous mixture. Hence, the optimum concentration of glutaraldehyde was determined at 5.6 × 10^−5^ mol. A previous study reported that cross-linking in chitosan causes increased mechanical properties. Meanwhile, the addition of plasticizers in the form of glycerol was intended to overcome the stiffness property of chitosan membranes.

**Figure 1:**
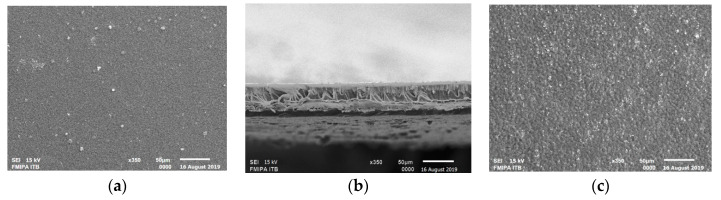
SEM Images (350× magnification) of the structure and morphology of the cross-linked chitosan/starch membranes made with a composition of 3% chitosan, 1.5% *D. hispida* starch, 5.6 × 10^−5^ mol glutaraldehyde, and 0.4% glycerol. (**a**) Top layer, (**b**) Cross-section, and (**c**) Bottom layer.

The FO membranes appeared yellowish, thin, and transparent, with strong physical characteristics, a dense porous structure, and a good FO performance [18]. The chitosan/starch FO membrane was revealed to have better characteristics and performance, compared to that made from neat chitosan. The chitosan/starch membrane had a thickness of 0.035 mm, a swelling degree of 28.98%, a porosity of 54.36%, a tensile strength value of 87.63 kgf/mm^2^, and an elongation of 16.08%. The chitosan/starch membrane had an asymmetric structure (as shown in Figure 1), where the top layer was thinner and tighter than the bottom layer. There were no macrovoid found in the membrane structure and the membrane had a stable interconnection, indicating strong mechanical properties [22]. In the FO test, this membrane had a water flux of 4.0 L/m^2^ h, where 1 M sucrose was employed as a DS.

### 3.3. Drinking Water Bags

**Figure 2:**
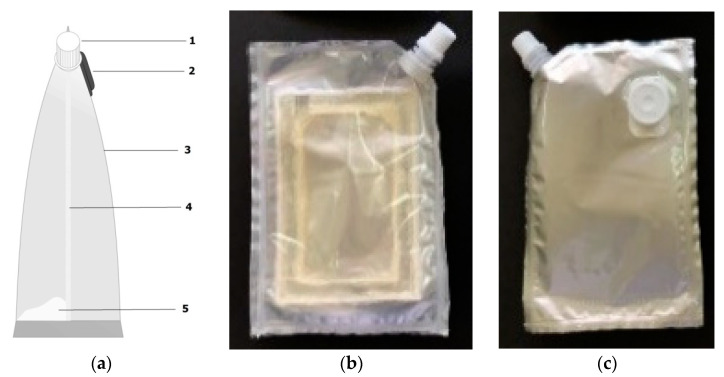
A drinking water bag made from a combination of aluminum foil and PP plastic with a chitosan/starch membrane filter inside. (**a**) Water bag design (1 = the lid on the front of the bag, 2 = the lid on the back of the bag, 3 = polypropylene plastic, 4 = chitosan-starch membrane, 5 = draw solution), with the photographs of (**b**) the front side and (**c**) backside of the bag.

The drinking water bags were made by referring to previous studies [9]; illustrated in Figure 2. The bag was made of three main materials, namely PP plastic, aluminum foil, and modified membrane. It is worth noting that the chitosan/starch membrane could not be laminated directly using a simple sealing machine because, based on the thermal analysis, the membrane material did not have a glass transition temperature [18]. After the moisture from the membrane material evaporated, the membrane was immediately observed to decompose at a temperature of 320 °C. 

Design of the drinking water bag was similar to commercially available FO filter bags. The three layers were arranged as follows—the PP plastic was assembled as the top layer, the membrane-attached PP plastic as the middle layer, and aluminum foil as the bottom layer. All three were glued altogether through lamination using a sealing machine, where the caps were added to the front and back sides, as shown in Figure 2. The manufactured drinking water bags had a total volume capacity of ±400 mL, with a volume capacity of ±200 mL in each side. The effective surface area of the membrane contact in this bag was 41.25 cm^2^.

### 3.4. Forward Osmosis Process

The water bags were used in FO testing with an FS of brackish and dam water. These feed solutions had different chemical characteristics (Table 1). Three types of DSs (fructose, sucrose, and their mixtures) were employed to investigate the chitosan/starch membrane’s performance in the water drinking bag. A total of 200 mL FS was and 100 mL DS was added to the back and front sides of the bag, respectively.

**Figure 3:**
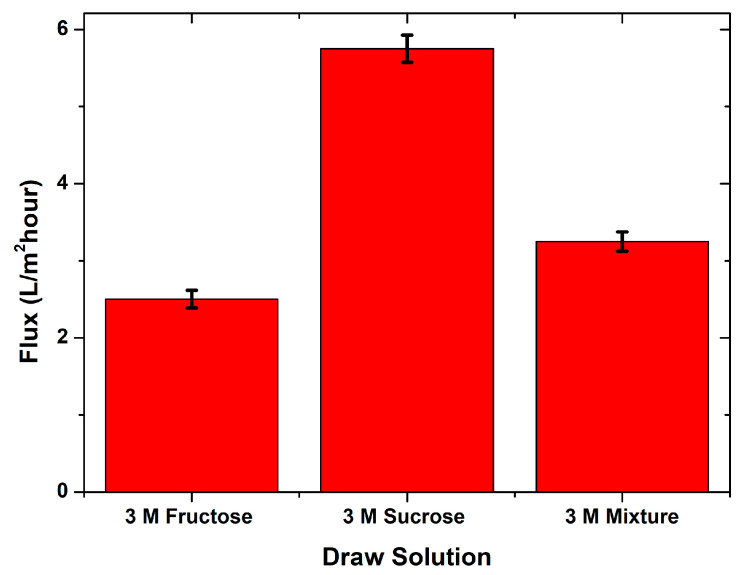
The water flux produced during the FO process brackish water as an FS with different DSs.

Figure 3 illustrates the water fluxes from the FO process using the three respective DSs. The optimum water flux was evident with a 3 M sucrose DS, which was 5.75 L/m^2^ h. This value was higher than in the previous study (5.25 L/m^2^ h), which employed an unmodified chitosan membrane and the same DS [9]. The cross-linking using glutaraldehyde was proven to improve membrane performance, where a better flux was observed. Lower water fluxes, 2.5 and 3.25 L/m^2^ h, were obtained from the fructose and the fructose/sucrose mixture DSs, respectively.

**Figure 4:**
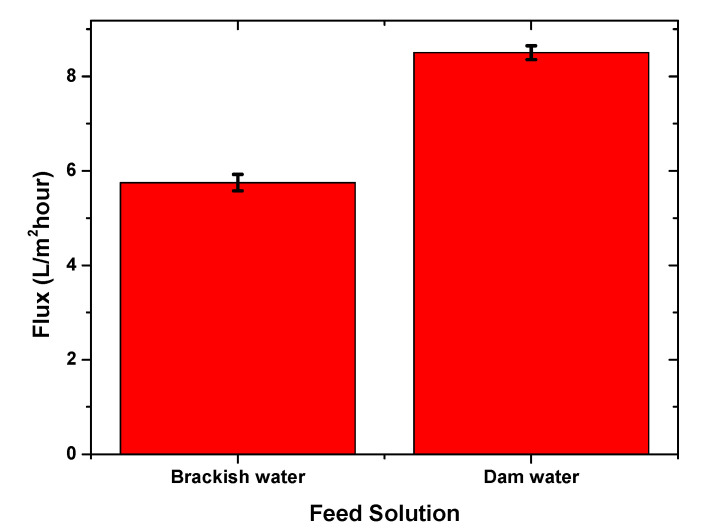
The resulting water flux used 3 M sucrose as a withdrawal solution with different FSs.

The use of sucrose as a DS was reported in several studies related to the FO process, in which it was found to generate a higher water flux compared to others [9,24,25,26,27,28]. This was ascribed to the higher osmotic pressure produced by the sucrose solution than fructose, glucose, and their mixture. The higher osmotic pressure of the draw solution led to a higher potential of the water flow permeation from the feed, which could be observed, based on the flux differences produced by fructose and sucrose. In this study, the chitosan/starch membrane was also evaluated using dam water; it was carried out with the same effective membrane surface area employing 3 M sucrose. Brackish water and dam water have different characteristics and produce different water flux values, as presented in Figure 4.

Figure 4 shows that the flux value generated from the dam water FS was higher (8.5 L/m^2^ h) than from brackish water (5.25 L/m^2^ h). The results could be associated with the feed water’s different characteristics, including pH, salinity, conductivity, and TDS. The dam water had lower ion contents than the brackish water; the more significant difference of concentrations between the feed solution and draw solution, and the greater the pressure produced led to a higher water flux [29].

**Figure 5:**
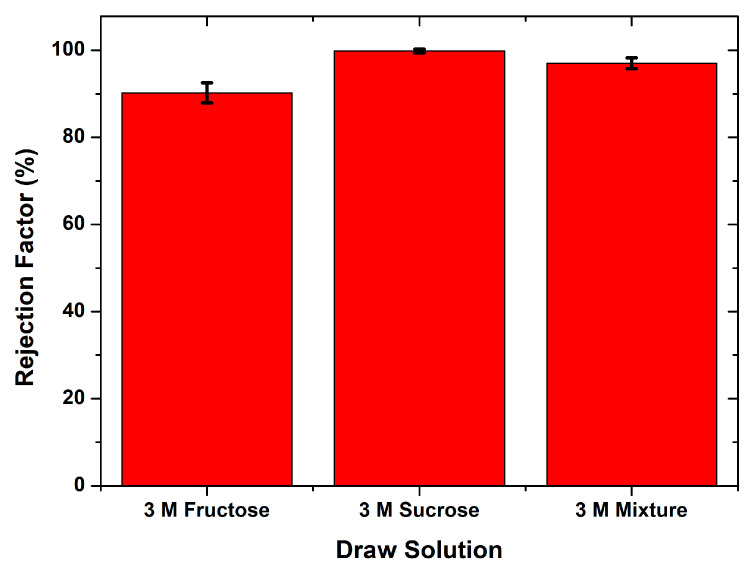
The rejection factor percentage of ion particles in the FO process from brackish water with different draw solutions.

In addition to the flux value, the chitosan/starch membrane performance could also be observed from the percent of a rejection factor value. Figure 5 shows the rejection percentage ranging from 90.2–99.8%. Based on the results, the percent rejection in this study showed a high separation of ion particles. The highest rejection percentage (99.8%) was obtained in the FO process from brackish water with a 3 M sucrose draw solution. On the contrary, the lowest percentage of rejection (92.2%) was obtained in the FO process with a 3 M fructose draw solution. The flux value and percent rejection factor obtained from the FO process in this study indicated that the modified chitosan membrane’s performance was better than the unmodified chitosan membrane, as reported in a previous study [9].

Based on the water flux and the rejection factor value, it could be seen that the changes in the membrane water flux affected the rejection factor of the membrane. Changing the draw solution from 3 M Fructose to 3 M sucrose increased the membrane water flux and simultaneously increased the rejection factor. The sucrose 3 M as a draw solution, the amount of water solvent that moved to the draw solution was increased and the feed solution became more concentrated. The salt concentration contributed to the rejection factor’s increase in using the 3 M sucrose draw solution. Increasing the water flux did not cause the transfer of dissolved ions in the feed solution to the draw solution. The same phenomenon was also observed in using a draw solution for a mixture of fructose and sucrose 3 M, where an increase in the water flux simultaneously resulted in a better rejection factor from the membrane.

### 3.5. FO Water Quality

Several water quality parameters was analyzed to assess the water quality of the Lamnyong River, Limpok village, Aceh Besar district. The main parameters related to brackish water quality are salinity, TDS, pH, conductivity, heavy metal content, and *E. coli* bacteria content. The results are shown in Table 1 and Table 2. According to the WHO [30] and Indonesian Health Ministry standards [31], several brackish water characteristics do not meet acceptable drinking water quality standards. According to the brackish water properties, the brackish water sample has a salinity value of 9 ppt, which indicates the dissolved salt contents. The presence of dissolved salts was also evidenced by the high TDS value of 1297 mg/L, attributed to the number of ions in the water. The high salinity value and dissolved ion contents in the water were expected to be separated by a forward osmosis membrane. However, low concentration heavy metal contents were observed in brackish water, which was still acceptable for quality drinking water and sanitation standards. All the metals analyzed (As, Cd, Cr, Cu, Fe, Hg, Mn, and Zn) were still relatively low in drinking water content. Moreover, the brackish water samples also tested negative for *E.coli* and *coliform* bacteria.

For cases where the parameters fell behind the quality standard, the water could be drinkable afterward by using our drinking water bag. The water would then contain sugar from the DS. The use of sucrose and fructose as a draw solution contributed to adding this drinking water bag’s practicality. The quality of the processed water was analyzed against the changes in all parameters of the brackish water. The following was the water quality produced after the FO process, carried out for one hour.

#### 3.5.1. Physicochemistry Water Properties

**Figure 6:**
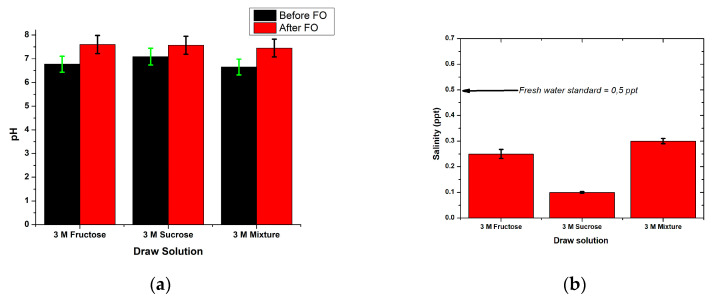

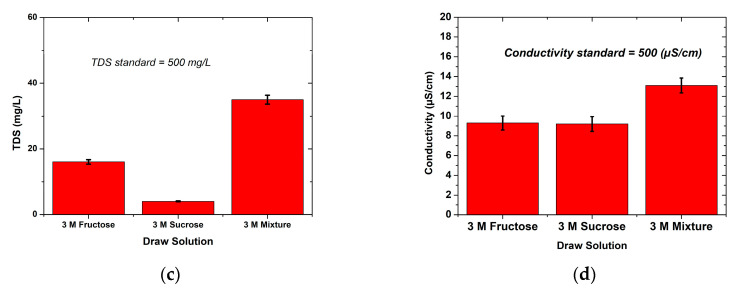
(**a**) pH, (**b**) salinity, (**c**) TDS, and (**d**) conductivity of the FO-processed water.

The water produced from the FO process was evaluated for pH, salinity, conductivity, and TDS, as shown in Figure 6. The draw solution’s pH value did not significantly change from before the FO process, ranging from 7.6–7.45 pH (Figure 6a). In line with these findings, the previous research reported that the FO process did not result in significant changes in pH values [5]. Hence, it could be expected that the pH values of the processed water, using the three DSs, were within the allowed range for drinking water quality standards (pH 6.5–8.5), according to the WHO [30] and the Indonesian government [32]. In addition to the pH, the salinity, conductivity, and TDS also showed promising results. The salinity of the FO-processed water (Figure 6b) ranged from 0.1 to 0.8 ppt. The lowest salinity (0.1 ppt) was obtained in the FO-processed water using 3 M sucrose DS. Meanwhile, the highest salinity value (0.8 ppt) was obtained from the water drawn using 3 M fructose. The salinity obtained from this study was lower than those in previous studies. It was reported that the salinity values obtained from FO with neat chitosan membranes were within the range of 0–1.3 ppt [5]. The chitosan/starch membrane could restrain salt particles better, as indicated by the lower salinity in the processed water. Based on these results, water produced by the FO process in this study was classified as freshwater with salinity levels that met the general quality standard (0.5 ppt) used for drinking water [33].

The water produced by the FO process in this study was also evaluated for TDS and conductivity to determine its ability to conduct electricity and also the number of dissolved solids present in the water. Figure 6c,d show the value of TDS and water conductivity resulting from the FO process. TDS values obtained were in the range of 2–116 mg/L and the conductivity values were in the range of 0.3–1.43 µS/cm. These results are known to be better than the TDS and conductivity values reported in previous studies [5]. It was reported that TDS and conductivity values after the FO process using an unmodified chitosan membrane ranged from 2–353 mg/L and 1.92–393 µS/cm. Based on these results, it was concluded that the modified chitosan membrane can hold particles better than the unmodified chitosan membrane. The maximum TDS value determined as the quality standard for drinking water by the WHO [30] was less than 600 mg/L.

#### 3.5.2. Heavy Metal Content

The FO process’s water was evaluated for the content of heavy metals As, Cd, Cr, Cu, Fe, Hg, Mn, and Zn, using an AAS. The content of heavy metals in water was essential because it is one of the WHO’s mandatory parameters in drinking water quality standards. The results of testing heavy metal content are shown in Table 2.

Table 2 above shows that the feed solution used in this experiment contained heavy metals in low concentrations. Thus, the change of heavy metal contents in the water after the FO process was also minimal. However, if the brackish water feed contained monovalent, divalent, or multivalent ions, based on the literature, it was possible to be retained by the chitosan-based FO [5]. The cross-linked chitosan/starch membrane in this study had a rejection factor above 90%, indicating a high ability to retain dissolved salt in brackish water. Most studies report high rejections of almost every type of heavy metal by FO-like membranes [32,34,35,36]. You et al. [37] reported that the FO membrane was able to reject heavy metals whose hydrated ion diameters were smaller than the membrane pore size; the charge–interaction should be responsible for heavy metal rejection. The FO water filtration membrane bags have N- and O-containing functional groups that allows heavy metal ions retention via electrostatic repulsion and chelation [38]. However, the FO’s heavy metal rejection could also be attributed to the role of the retained multivalent anions from the FS in maintaining the electroneutrality [39]. High diffusion during the FO process, as a consequence of maintaining the DS’s electroneutrality, led to a counter ion transfer. Hence, ion exchange between the two solutions did not occur, proven by our FO-processed water’s low ion contents.

#### 3.5.3. Biological Water Properties

Other than being beneficial to humans, water is also a good medium for bacterial life. Bacteria are divided into two categories, namely pathogenic and non-pathogenic bacteria. Pathogenic bacteria can cause disease and diarrhea. Clean water that is safe to drink must meet the requirements set by the government and WHO. One of these standards relates to the microbiological conditions, where the *E. coli* should not be found in 100 mL of the water. *E.coli* itself is one of the pathogenic bacteria.

Saiful et al. reported that the chitosan-based drinking water bags are able to filter all types of *E. coli* and coliform bacteria [5,9]. *Escherichia coli* is a rod-shaped bacterium. Each of the bacteria was measured approximately to be 0.5 μm in width by 2.0 μm in length. Its dimensions are those of a cylinder 1.0–2.0 μm long, with a radius of about 0.5 μm [40,41]. *E. coli* and coliform bacteria have a larger size and are retained by the chitosan-dense membrane; bacteria from the FS side cannot pass through the membrane to the DS side. Moreover, chitosan is well-known for its antibacterial properties, by changing the permeability of the bacterial cell wall. It allows the bacteria to be killed on the membrane surface, leading to its application in anti-biofouling membranes [42]. However, based on the test results from the Aceh Health Laboratory Aceh Province and FMIPA Chemistry Laboratory, Department of Chemistry, Syiah Kuala University, the brackish water used in the FO process did not contain *E. coli* and coliform bacteria. As a result, the contribution of the FO process carried out by the drinking water bag in eliminating the *E. coli* was insignificant. Nonetheless, the process could be very helpful to sterilize the water from microbial content during an emergency, where the level of contamination was expected to be high [43].

### 3.6. FO Filter Bag Durability

As a filter bag, this cross-linked chitosan/starch filter bag has some advantages despite the facts that there are always more room for improvement. First, with chitosan and starch raw materials available in nature that can be processed quickly, the price of drinking water bags can be competitive with existing products in the market. Moreover, with regards to membrane lifetime, as a drinking water bag is mostly made for single-use, the chitosan/starch membrane used in this study can be stored for a relatively long time. In a preliminary study, the FO drinking water bag did not change and leaked for about two weeks, which was indicated by no change in the TDS of the feed solution and the withdrawal solution, after reaching an equilibrium osmotic pressure that led to zero net flux. 

## 4. Conclusions

This study indicated that the modified chitosan membrane was successfully applied to manufacture drinking water bags for the FO process. The different flux values produced in this study were attributed to the type of draw solution and ion content in the FS. The highest water flux was generated by the FO-drinking water bag using 3 M sucrose DS with values of 5.75 L/m^2^ h and 8.5 L/m^2^ h for brackish water and dam water FSs, respectively. The percentage of rejection produced in this study also showed a positive performance of the modified chitosan membrane, with a 99.8% value. This research also showed that the modified chitosan membrane was indicated to be successful in retaining salt, metals, bacteria, and other solutes. During the FO process, the filtered water met the WHO’s quality standards set for drinking water. Based on these results, it can be concluded that the modified chitosan membrane has the potential to be used as an alternative water purification process in emergency water purification in order to produce drinking water containing sugar as an energy source for the body.

## Figures and Tables

**Table 1:** The quality of the feed water used in the FO process.

Sample	Parameter			
	pH	Salinity (ppt)	Conductivity (µS/cm)	TDS (mg/L)
Brackish Water	7.91	9.3	15.83	1297
Dam Water	7.19	0.1	0.39	27

**Table 2:** The results of the analysis of heavy metal contents in brackish water and FO-processed water.

No	Parameter	Metal Content (mg/L) FO Product Water				Standard (mg/L)
		Brackish Water	Sucrose	Fructose	Mixture	
1	Mercury (Hg)	<0.001	<0.001	<0.001	<0.001	0.006
2	Arsenic (As)	<0.003	<0.003	<0.003	<0.003	0.01
3	Zinc (Zn)	<0.01	<0.01	<0.01	<0.01	3
4	Copper (Cu)	<0.0007	<0.0007	<0.0007	<0.0007	2
5	Chromium (Cr)	<0.002	<0.002	<0.002	<0.002	0.05
6	Iron (Fe)	0.1071	0.0634	0.0214	0.0219	0.2
7	Cadmium (Cd)	<0.002	<0.002	<0.002	<0.002	0.003
